# Grazing Seasons and Stocking Rates Affects the Relationship between Herbage Traits of Alpine Meadow and Grazing Behaviors of Tibetan Sheep in the Qinghai–Tibetan Plateau

**DOI:** 10.3390/ani10030488

**Published:** 2020-03-15

**Authors:** Xiang Xiao, Tao Zhang, Jay Peter Angerer, Fujiang Hou

**Affiliations:** 1 State Key Laboratory of Grassland Agro–ecosystems, College of Pastoral Agriculture Science and Technology, Lanzhou University, Lanzhou 730020, China; xiaox17@lzu.edu.cn; 2 Key Laboratory of Grassland Livestock Industry Innovation, College of Pastoral Agriculture Science and Technology, Lanzhou University, Lanzhou 730020, China; 3 Ministry of Agriculture and Rural Affairs; Lanzhou University, College of Pastoral Agriculture Science and Technology, Lanzhou 730020, China; 4 Institute of New Rural Development, Guizhou University, Guiyang 550025, China; 5 Texas A&M AgriLife Research, Blackland Research and Extension Cent, 76502–9622, Temple, TX 76502, USA; jangerer@brc.tamus.edu

**Keywords:** rotational grazing, herbage mass, dry matter intake, walking velocity, crude protein, rumination, metabolic energy

## Abstract

**Simple Summary:**

The relationship between vegetation and grazing behavior of Tibetan sheep on the Qinghai–Tibetan Plateau (QTP) remains a major concern for pursuing the sustainable grazing management of grassland. Grazing behavior is the daily activity of grazing livestock, which can reflect the growth status of the pasture and the level of grassland health in the local pasture, as well as the nutritional needs of Tibetan sheep. We studied the relationship between the grazing behaviors of Tibetan sheep and the quantity and quality of forage in different seasons and different stocking rates. Our results showed that the grazing behavior of Tibetan sheep was greatly affected by the quantity and nutritional quality of the forage. These results may be helpful for local herders to evaluate the nutritional status of forage and condition of grassland degradation, so that appropriate measures can be taken to protect local pastures in advance.

**Abstract:**

Under the combined effect of stocking rate and grazing season, it is very significant to ascertain whether there is a quantitative relationship between plant community characteristics, chemical composition of forage, and grazing behaviors of Tibetan sheep to better utilize native pasture in the northeast region of the Qinghai–Tibetan Plateau (QTP). The two consecutive year observation experiments on Tibetan sheep’s grazing behavior were conducted to evaluate the above-stated relationships between stocking rates of 8 sheep/ha and 16 sheep/ha stocking rates in the both the warm and cold seasons. The results demonstrated that at 8 sheep/ha or in the warm season, due to better forage quality, Tibetan sheep had higher herbage mass, forage crude protein (CP) concentration, CP intake, dry matter intake (DMI), and interval between feed boluses and total number of steps, as well as lower fiber concentration than that at 16 sheep/ha or in the cold season. Diurnal intake rate and walking velocity while intaking increased as both average daylight ambient temperature and relative humidity rose. Using the CP concentration, acid detergent fiber (ADF) concentration, neutral detergent fiber (NDF) concentration, and forage metabolic energy (ME) to predict grazing behavior yielded the best fit equation for Tibetan sheep. For local herdsmen to sustainably use the alpine meadow, 8 sheep/ha in the warm season should be considered as the better grazing condition for preventing grassland degradation.

## 1. Introduction

Livestock grazing behavior plays a key role in the interactions between pasture and animals, which is a central factor in animal production and grassland community structure [1,2]. Grazing behavior is influenced by a range of factors such as climate, sward height, herbage mass, botanical composition of pastures, desirable species, forage quality [3,4], age [5], breed [6], animal management [7], and grazing management [8]. Grazing management (e.g., grazing season and stocking rate) affects pasture growth [9], which, in turn, affects livestock grazing behavior [4]. Furthermore, the grazing behaviors of livestock change at different stocking rates. For instance, on the Qinghai–Tibetan Plateau (QTP), it has been shown that the daylight grazing time increased but ruminating time decreased for Tibetan sheep as a result of an increase in grazing intensity [10]. Grazing time of sheep increased and time of rest decreased with increasing grazing intensity in the Inner Mongolian steppe, China, as a result of reduced forage availability at the higher grazing intensity [11]; similar results have been found for cattle in Florianópolis, Brazil [12]. Simultaneously, livestock grazing behavior responds to the phenological period of pasture vegetation because of variations in herbage mass, forage quality, and daylight duration [10]. On the QTP, the decrease in intake time, ruminating time, number of chews per feed bolus, walking distance per hectare, and daily walking distances in winter are most likely due to the harsh weather conditions and the decline in forage quality [10]. The quality of forage affects herbivores’ feed intake, so nutritional needs are maintained [13]. Furthermore, the structure and composition of plant communities are affected by the selective feeding of livestock [14,15].

However, few studies focus on the change of grazing behavior under different grazing seasons and stocking rates, and our understanding of the relationship between grazing behavior of livestock and grassland condition remains vague. To quantitatively estimate whether the effects of grazing season and stocking rate on feed behavior are due to changes in the plant community characteristics and chemical composition of forage, a rotational grazing trial of Tibetan sheep has been implemented with different stocking rates and grazing seasons on QTP. We hypothesize that: (1) at lower stocking rate and in the warm season, total steps of Tibetan sheep would decrease, due to the greater forage allowance and thereby more available intake selectivity in the growing season; (2) intake rate and walking velocity while intaking increases linearly with increasing temperature and humidity; (3) dry matter intake (DMI) of Tibetan sheep may increase as the stocking rate rise or the grazing season changes. The results of the present study may contribute to our understanding of the interaction mechanism between plants and animals and may provide a theoretical basis for the rational use of resources and the reasonable management of the ecosystem in alpine meadows on the QTP. 

## 2. Materials and Methods

### 2.1. Study Area Description

**Figure 1:**
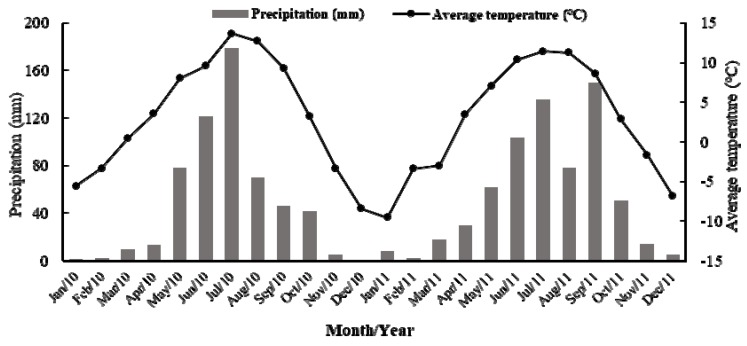
Mean monthly precipitation and temperature from January 2010 to December 2011 at the experimental site located in an experimental pasture farm in Maqu County (35°58’N; 101°53’E) on the Qinghai–Tibetan Plateau (QTP). Temperature: average 2.9 °C; high 13.7 °C; low 9.5 °C. Rainfall: average 50.8 mm; high 178.8 mm; low 0.5 mm (Data from Maqu County Meteorological Station).

Field trials were conducted in the Maqu Grassland Agriculture Trial Station of Lanzhou University in Maqu County, Gansu Province, China (35°58′N; 101°53′E; altitude 3700 m). The average annual temperature is 1.2 °C and the average annual rainfall is 620 mm, of which 83.8% mainly takes place from May through to September (Figure 1). The grassland is classified as the “alpine meadow” [16] and the soils are classified as Mat–Cryic Cambisols soil [17]. Alpine meadow in the study site grows from late April to early September. The dominate species include: Cyperaceae (mainly *Kobresia*, Willd.; *Carex*, L.), Gramineae (mainly *Stipa*, L.; *Festuca*, L.; *Elymus*, L; and *Poa*, L.), Compositae (mainly *Saussurea* DC.), Ranunculaceae (mainly *Anemone*, L.), and other Forbs (mainly *Polygonum viviparum* L. and *Potentilla anserina* L.).

### 2.2. Design of the Grazing Experiment

The experiment was conducted in early June to early September (warm season) and early October to late December (cold season) in both 2010 and 2011. In early May, 150 1.5–year–old Tibetan rams (about 20% for substitution of the dead and sick sheep) were bought and were sold after winter grazing trial every year.

In each grazing season, plots were laid out in a random block design with six total plots for each stocking rate, which were randomly arranged in two rows and six columns of test areas. Eight sheep were allocated to each plot, and three plots were randomly selected from six plots of each stocking rate and fixed for observation of grazing behavior.

The size of the plots was used to control the stocking rate so that the number of animals would be the same across stocking rate treatment levels. Therefore, the high stocking (16 sheep/ha) plots were 0.5 ha each and the low stocking rate (8 sheep/ha) plots were 1 ha each. Thus, there were four treatments (warm season and 8 sheep/ha, W8; warm season and 16 sheep/ha, W16; cold season and 8 sheep/ha, C8; cold season and 16 sheep/ha, C16) in every year.

Before the experiment, firstly, the weight data of all sheep were statistically analyzed, and there were no significant differences among the groups. Each sheep was numbered according to each group of grazing paddocks and was then assigned to the fixed paddocks. We used the special sheep balance to weigh the Tibetan sheep once a month. The technical information of the balance is as follows: the electronic floor scale is surrounded by a fence with the length of 0.6 m, the width of 0.5 m, and the height of about 10 cm from the ground; the accuracy is 0.5 kg, the load range is 0–1000 kg, the steel plate thickness is 4 mm, the self–weight is about 20 kg, the dynamic weighing accuracy range is 1%–6%, and the maximum safety load is 150%. The sensor uses the international advanced integrated sensor, which is equipped with a temperature compensation circuit and a dual signal circuit. The initial bodyweight (IBW) and average daily weight gains (ADGs), respectively, of Tibetan sheep were as follows: W8, 31.899 ± 1.196 and 0.138 ± 0.021 kg; W16, 31.393 ± 1.447 and 0.124 ± 0.011 kg; C8, 46.846 ± 1.213 and 0.039 ± 0.010 kg; C16, 43.974 ± 1.423 and 0.031 ± 0.012 kg.

In the warm season (July 1 to September 30), animals grazed the pastures each day at 08:00 and returned to the pens at 19:00, and the total time allowed for grazing was 660 min/day. In the cold season (October 1 to December 31), animals went into the pastures at 09:30 and returned to the pens at 16:30, and total grazing time was 420 min/day. Each group of sheep returned to the fixed pen to stay overnight for safety. Each day, sheep were allowed to drink water with salt freely after coming back to the pen. In each paddock, there were 10 days grazing followed by a 20–day grazing interval.

### 2.3. Sward Measurement

First, three 0.5 × 0.5 m^2^ squares were randomly selected on the diagonal of each grazing plot, after which all plants were measured with a ruler to calculate mean sward height, and the quantity of each plant was counted to assess mean plant density after 10 days of grazing. Then, herbage mass was measured. For this, a fresh sample was obtained by cutting all of the herbage to ground level inside the three squares (0.5 × 0.5 m^2^) randomly selected in each grazing plot. The sample was then placed in an oven at 60 °C for 48 h. Finally, dry samples of forage were crushed through a 1 mm sieve and packed in clear plastic ziplock bags for analysis of nitrogen (N), acid detergent fiber (ADF) concentration, and neutral detergent fiber (NDF) concentration. The N concentration in the sample was determined as described by Ma et al. [18] and then multiplied by 6.25 to obtain crude protein (CP) concentration. The NDF and ADF concentrations were determined as described by Goering and Van Soest [19] and analyzed sequentially using an ANKOM 2000 fiber analyzer (ANKOM Technology, Fairport, NY, USA). The organic matter (OM) concentration was determined from the difference between dry mass and ash concentration [20].

### 2.4. Fecal Sample Collection

The fecal samples of the three selected Tibetan sheep in each grazing paddock were collected into fecal bags for 3 consecutive days during the grazing period, then dried at 60 °C for 48 h and weighed. The organic matter (OM) concentration in feces was measured from the difference between fecal dry matter and ash concentration in feces [20]. The data were used for the calculation of dry matter digestibility (DMD) [21].

### 2.5. Observation of Animal Grazing Behavior

During the grazing trials, three of the eight male Tibetan sheep in each grazing plot were randomly selected and marked with a colorful ribbon to allow identification from a distance. The grazing behavior of the Tibetan sheep was recorded by three trained observers, who were located at least 10 m away from the sheep, by observing foraging and movement with telescopes and assessing the amount of time spent conducting a particular activity using stopwatches. The grazing behavior was continuously observed for 3 days and recorded for 15 min, one time per hour [10,22]. The three observers were randomly allocated for one high and one low stocking rate of the paddock in each season of grazing land, and they were responsible for the two fixed grazing paddocks during the experiments. 

The observed indicators included the following [22]: diurnal intake time (min/day), the amount of time that Tibetan sheep spent on intaking forage in a day; intake rate (bites/min), the average number of bites taken for intaking forage in five minutes; walking velocity while intaking (steps/min), the average number of steps in five minutes (including forward and backward) while Tibetan sheep are intaking forage; bite of ingestion (bite/step), the average number of bite per step; bite mass (g/bite); total number of steps (step/day); number of chews per feed bolus (times), the average number of chews for chewing five boluses; chewing time per bolus (s), average time while chewing five boluses; interval time between feed boluses (s), the average interval time between chewing each of the five boluses; and ruminating rate (bolus/h), the average number of boluses for chewing in each period of rumination. We measured the bite weight through simulating the Tibetan sheep’s intaking area, height, and species of the plant.

### 2.6. Calculation Formula 

Calculations were made to estimate DMI (g/day per sheep) as follows:

(1) $$DMI=\frac{D\times S}{N}$$

(2) $$D=\frac{\left(A-B)\times (\log C-\log B\right)}{\log A-\log B}$$

where S is the area of the grazing plot, N is the number of Tibetan sheep in the grazing plot, D is a is the herbage consumption (g/m^2^) during 5 days of grazing, A is the herbage mass (g/m^2^) before grazing, B is the herbage mass (g/m^2^) after 5 days of grazing, and C is the herbage mass (g/m^2^) in a cage (1 × 1 × 1 m^3^) after 5 days of grazing [23].

Calculations were made to estimate crude protein intake (CPI, g/day) as follows:

(3) CPI=CP×DMI

Calculations were made to estimate dry matter digestibility (DMD, %) as follows:

(4) $$DMD=\frac{DMI-FDM}{DMI}\times 100\%$$

where DMD is dry matter digestibility and FDM is fecal dry matter. 

Calculations were made to estimate forage metabolic energy (ME, MJ/kg DM) [24] as follows:

(5) ME=0.0157×DOMD

(6) DOMD=DOM/DM

(7) DOM=DMI×OM−FDM×OMF

where ME is the forage metabolic energy, DOMD (g/kg DM) is the digestible organic matter in the dry matter [25], DOM (g) is the digestible organic matter, DM (kg) is the dry matter, OM (%) is the organic matter in forage, and OMF (%) is the organic matter in feces. 

Calculations were made to estimate metabolic energy intake (MEI, MJ/day) as follows:

(8) $$MEI=k_{1}BW^{0.75}+ME_{g}\cdot ADG$$

where the k_1_ values (MJ/kg^0.75^) were 0.45 from July to September, 0.50 from October to November, and 0.55 in December. The values chosen for k_1_ were from the work of Koong et al. [26] and Degen and Young [27]. ADG is the average daily bodyweight gain (kg/day), and ME_g_ is the amount of ME required to gain 1 kg bodyweight and was taken as 30 MJ/kg [28].

### 2.7. Statistical Analysis

Statistical analyses were performed with the SPSS software (version 20.0). *F*–tests or differences in means were considered statistically significant at *p* < 0.05. Data on plant community characteristics, chemical composition of forage, digestive and metabolic indicators, and grazing behavior (except intake rate, walking velocity while intaking, and bite of ingestion) of Tibetan sheep affected by stocking rate and grazing season were analyzed using mixed–effect model:

Y=SR+GS+SR·GS+(1|Year/GS_unit/Plot)

which is a mixed–effect model with fixed effects (SR, GS, SR×GS) and random effects (Plot nested within GS_unit, and GS_unit nested within Year). Tukey’s test was used to evaluate differences among means and the significance level was set at 0.05.

Data on intake rate, walking velocity while intaking, and bite of ingestion affected by stocking rate, grazing season and time were analyzed using mixed–effect model:

Y=SR+GS+Time+SR·GS+SR·Time+GS·Time+SR·GS·Time+(1|Year/GS_Plot/Animal)

which is a mixed–effect model with fixed effects (SR, GS, SR×GS) and random effects (Plot nested within GS_unit, and GS_unit nested within Year). Tukey’s test was used to evaluate differences among means and the significance level was set at 0.05.

The data of diurnal intake rate and walking velocity while intaking were obtained from the two stocking rates (8 sheep/ha and 16 sheep/ha) in the warm and cold seasons of 2010 and 2011, which were used to evaluate their effects of daylight ambient temperature and relative humidity by linear regression. Using SPSS software (version 20.0), linear regression analysis was carried out to yield prediction equations of unary linear regression, as shown below:

Y=a+bx

where Y is the value of intake rate or walking velocity while intaking, ɑ is a constant term, x is the independent variable, which represents daylight ambient temperature or relative humidity, and b is Y corresponding partial regression coefficients for x. The effect of the linear term of each individual predictor variable was significantly different at the 0.05 level. The comparison was made between every two regression lines using the SPSS software to test for significant differences in overall intercept and overall slope.

Structural equation modeling (SEM) [29] was used to identify the relationship between grazing behavior and vegetation condition. In the model, we considered all plausible pathways (sward height, herbage mass, CP, ME, intake rate, bite weight, diurnal intake time) through which grazing season and stocking rate influence the DMI. The standard path coefficients were estimated to indicate the strengths of these multiple effects. The overall fit of the model was evaluated using the chi–squared test, the root–mean–square error of approximation (RMSEA) and the goodness of fit index (GFI). Non–significant χ^2^, RMSEA (*p* > 0.05), and GFI > 0.90 indicate a good fit of the model. SEM analysis was performed using AMOS 17.0 for Windows (Amos Development Company, Greene, Maine, USA; SPSS Inc., Chicago, IL, USA). 

To evaluate the effect of plant community characteristics and the chemical composition of forage on grazing behavior of Tibetan sheep, using SPSS software (version 20.0), step–wise multiple linear regression analysis with backward elimination was carried out to yield prediction equations of linear regression, as shown below:

$$Y=a+b_{1}x_{1}+b_{2}x_{2}+b_{3}x_{3}+\cdots +b_{n}x_{n}$$

where Y is a grazing behavior parameter, ɑ is a constant term, x_1_, x_2_, x_3_, …, x_n_ are independent variables that represent plant community characteristics (sward height, herbage mass) and the chemical composition of forage (CP, ADF, NDF and ME), and b_1_, b_2_, b_3_, …, b_n_ are Y corresponding partial regression coefficients for x_1_, x_2_, x_3_, … x_n_. The effect of the linear term of each individual predictor variable was significantly different at the 0.05 level.

## 3. Results

### 3.1. Plant Community Characteristics and Chemical Composition of Forage

The season and stocking rate had significant effects on sward height, plant density, herbage mass, ADF, NDF, and CP (*p* < 0.05; Table 1). The interaction between grazing season and stocking rate had significant effects on NDF and CP (*p* < 0.001).

**Figure 2:**
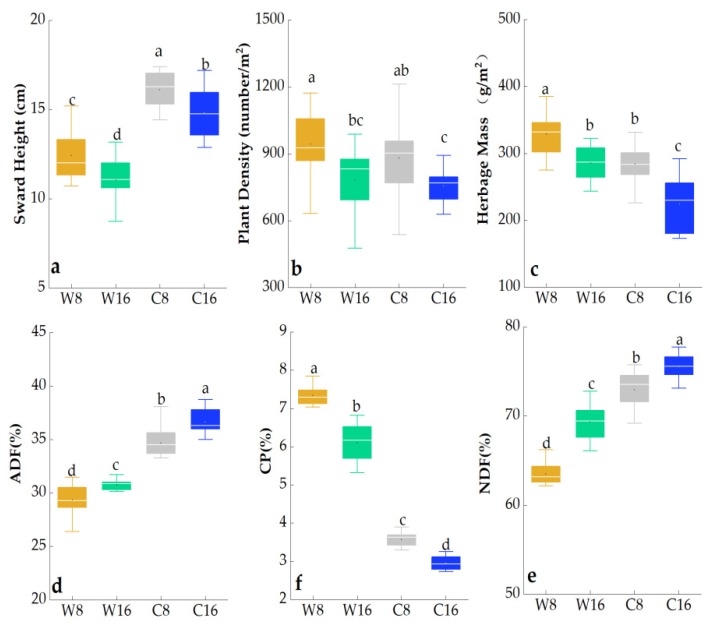
Effects of grazing seasons (warm and cold) and stocking rates (8 sheep/ha and 16 sheep/ha) on mean (± standard error (SE)) plant community characteristics and chemical composition in the two years (2010 and 2011). Means within a column with different letters significantly differ (*p* < 0.05). Different lowercase letters indicate significant differences.

The sward height, plant density, herbage mass, and CP concentration at 8 sheep/ha were significantly higher than those at 16 sheep/ha, but ADF and NDF concentrations at 8 sheep/ha were significantly lower than those at 16 sheep/ha. Compared with the cold season, sward height, herbage mass, and CP concentration were higher in the warm season, but sward height, ADF, and NDF concentrations were lower (Figure 2)

### 3.2. The Digestive and Metabolic Indicators of Tibetan Sheep under Different Stocking Rates and Grazing Seasons

**Figure 3:**
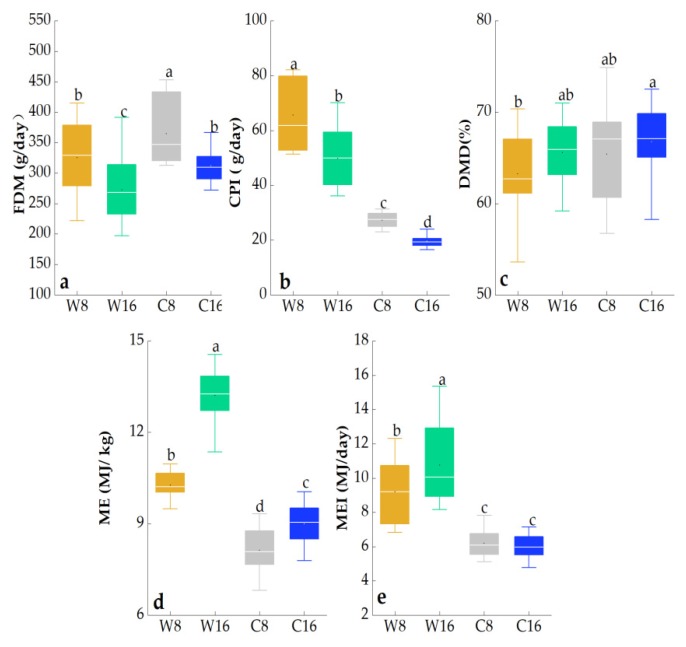
Effects of grazing seasons (warm and cold) and stocking rates (8 sheep/ha and 16 sheep/ha) on mean (± SE) digestive and metabolic indicators in two years (2010 and 2011). Means within a column with different letters significantly differ (*p* < 0.05). Different lowercase letters indicate significant differences.

The season and stocking rate had highly significant effects on FDM, CPI, and ME (*p* < 0.001; Table 2). The interaction between grazing season and stocking rate had significant effects on CPI and ME (*p* < 0.001). The FDM and CPI at 8 sheep/ha was significantly higher than that at 16 sheep/ha, but ME and MEI in the warm season at 8 sheep/ha were significantly lower than that at 16 sheep/ha. Compared with the cold season, CPI and ME were higher in the warm season, but FDM was lower in the warm season (Figure 3).

### 3.3. Diurnal Dynamics of Feeding Behavior

The grazing season and stocking rate had highly significant effects on intake rate and walking velocity while intaking (*p <* 0.001). Time significantly influenced intake rate (*p <* 0.001), and the interaction between grazing season and time significantly influenced intake rate and walking velocity while intaking (*p <* 0.001). The grazing season, stocking rate, time, and their interaction had highly significant effects on bite of ingestion (*p <* 0.05). 

**Figure 4:**
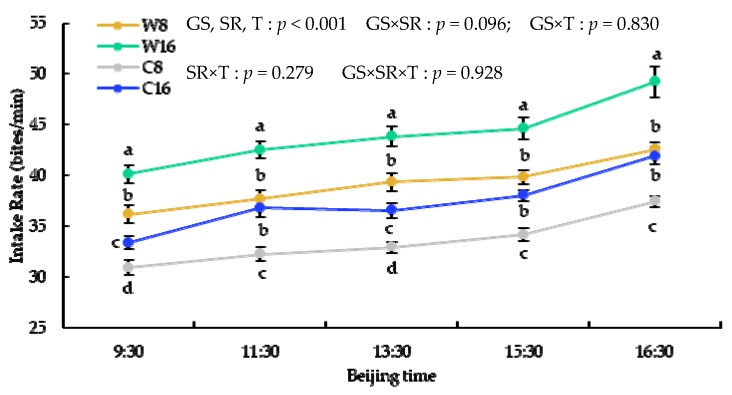
The x–axis is the time of day, and the y–axis is the intake rate. GS = grazing season; SR = stocking rate; T = times.

**Figure 5:**
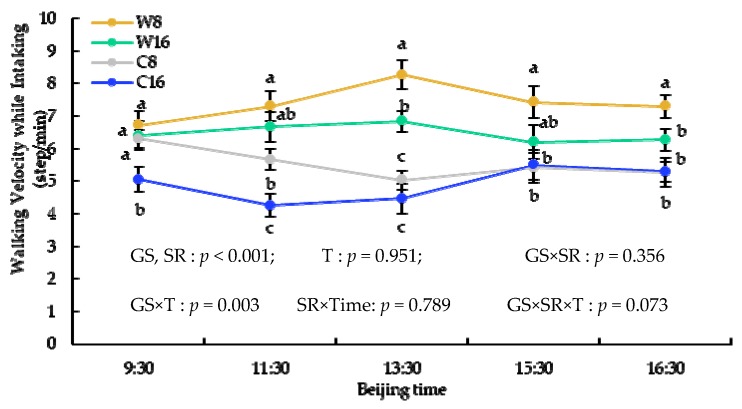
The x–axis is the time of day, and the y–axis is walking velocity while intaking. GS = grazing season; SR = stocking rate; T = times.

**Figure 6:**
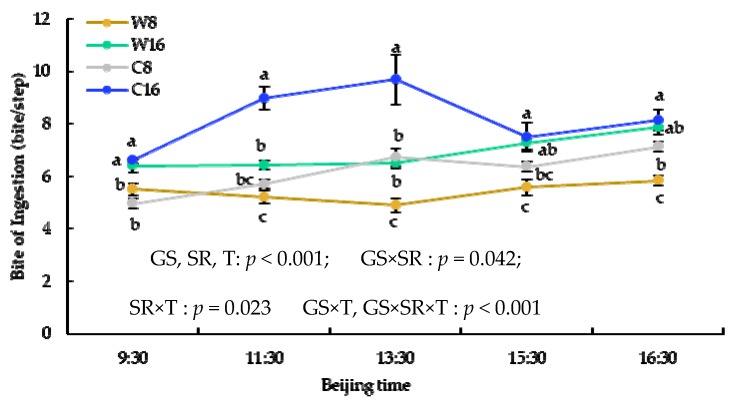
The x–axis is the time of day, and the y–axes is the bite of ingestion. GS = grazing season; SR = stocking rate; T = times.

The intake rate and bite of ingestion had an upward trend over time and were significantly higher at 16 sheep/ha than that at 8 sheep/ha, but walking velocity while intaking at 8 sheep/ha was higher than that at 16 sheep/ha in almost every time period during the grazing. The intake rate and walking velocity while intaking in the warm season were significantly higher than that in the cold season, but bite of ingestion had an opposite trend (Figure 4, Figure 5 and Figure 6).

### 3.4. Grazing Behavior of Tibetan Sheep under Different Stocking Rates and Grazing Seasons

The grazing season had highly significant effects on diurnal intake time, bite weight, DMI, total number of steps, number of chews per feed bolus, chewing time per feed bolus, interval between feed boluses, and ruminating rate (*p <* 0.05; Table 3). Stocking rate had highly significant effects on diurnal intake time, bite weight, DMI, number of chews per feed bolus, chewing time per feed bolus, and ruminating rate (*p <* 0.01). The interaction between grazing season and stocking rate significantly influenced bite weight (*p <* 0.001).

**Figure 7:**
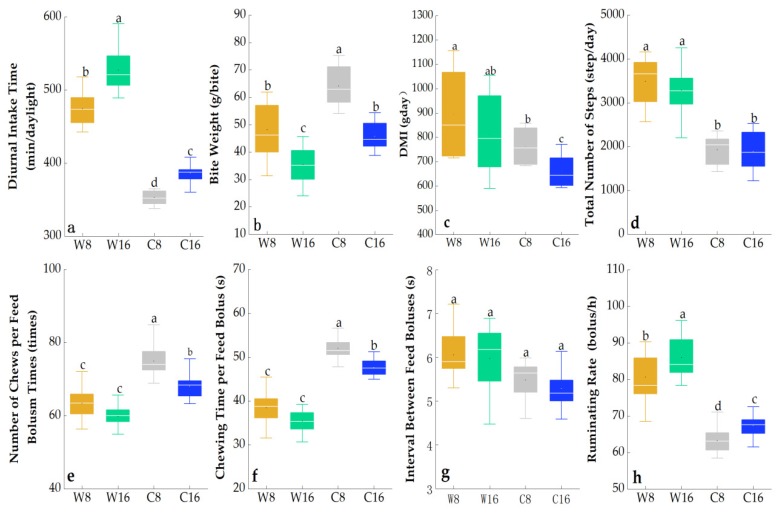
Effects of grazing season (warm and cold) and stocking rate (8 sheep/ha and 16 sheep/ha) on mean (± SE) gazing behavior in the two years (2010 and 2011). Means within a column with different letters significantly differ (*p <* 0.05).

In general, compared with a stocking rate of 8 sheep/ha, bite weight, DMI, number of chews per feed bolus, and chewing time per feed bolus were lower at 16 sheep/ha, while diurnal intake time and ruminating rate were higher. Compared with the cold season, diurnal intake time, DMI, total number of steps, and ruminating rate were higher in the warm season, while bite weight, number of chews per feed bolus, and chewing time per feed bolus were lower (Figure 7). 

### 3.5. Relationship Between Ambient Temperature, Relative Humidity, and Intake Behavior

**Figure 8:**
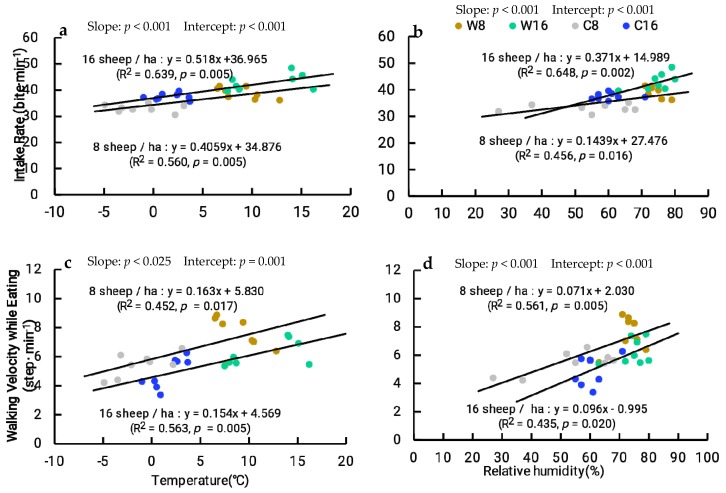
Relationships between (**a**) average daylight ambient temperature and intake rate, (**b**) relative humidity and intake rate, (**c**) average daylight ambient temperature and walking velocity while intaking, (**d**) relative humidity and walking velocity while intaking at the two stocking rates (8 sheep/ha and 16 sheep/ha) in the two seasons of the (warm and cold) of 2010 and 2011. The slope and intercept in the figure represent the difference between the two regression lines.

Because there are statistical differences in the values of the intercepts and the slopes, every two regression lines are different (Figure 8). There was higher intake rate and lower walking velocity while intaking at 16 sheep/ha than that at the 8 sheep/ha. As daylight ambient temperature increased, intake rate and walking velocity while intaking had an upward trend at 8 and 16 sheep/ha stocking rates. With the increase of relative humidity, intake rate and walking velocity while intaking also increased (Figure 8).

### 3.6. Structural Equation Model

**Figure 9:**
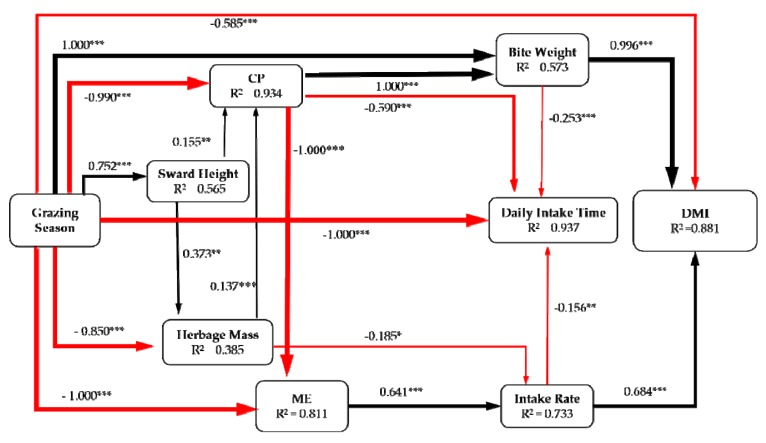
Structural equation modeling (SEM) showed the effect of grazing season on dry matter intake (DMI). The evaluation for grazing season: warm season = 1, cold season = 2. The SEM considered all plausible pathways (sward height, herbage mass, crude protein (CP), metabolic energy (ME), intake rate, bite weight, diurnal intake time) through which stocking rate influences the DMI. The arrows reflect the causal relationships, and the thickness of the black (positive) and red (negative) paths is proportional to the range–standardized path coefficients. * *p <* 0.05, ** *p <* 0.01, *** *p <* 0.001. The model shows a fitted result (χ^2^ = 0.432, df = 13, *p* = 0.934; RMSEA = 0.053, *p* = 0.651; GFI > 0.986).

The SEM was used to evaluate the potential associations between plant community characteristics, the chemical composition of forage, feeding behavior, and DMI, and to gain insight into the indirect and direct effects. The grazing season explained 93.4%, 56.5%, 38.5%, 81.1%, 73.3%, 57.3%, 93.7%, and 88.1% of the variation in CP concentration, sward height, herbage mass, ME, intake rate, bite weight, diurnal intake time, and DMI, respectively (Figure 9). The change of grazing season (from warm season to cold season) reduced herbage mass, CP, ME, diurnal intake time, and DMI but had positive effects on sward height and bite weight. However, sward height had a positive effect on herbage mass and CP, while herbage mass had a negative effect on intake rate and a positive effect on CP, and ME positively affected intake rate. CP had a positive effect on bite weight and negative effects on ME and diurnal intake rate. Furthermore, intake rate and bite weight both had positive effects on DMI (Figure 9).

**Figure 10:**
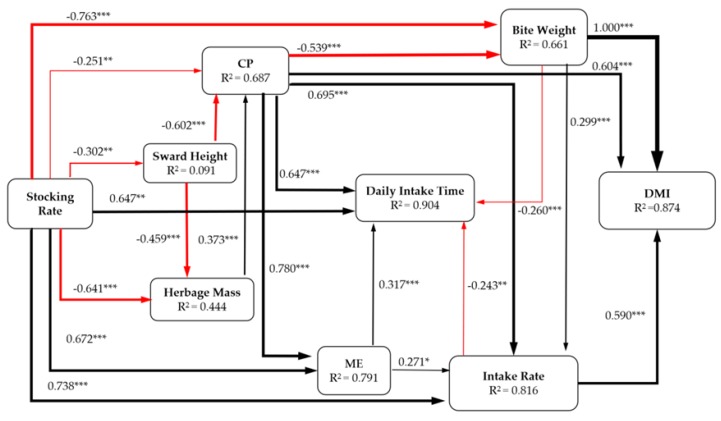
The SEM showed the effect of stocking rate on DMI. * *p <* 0.05, ** *p <* 0.01, *** *p <* 0.001. The model shows a fitted result (χ^2^ = 10.770, df = 12, *p* = 0.459; RMSEA = 0.051, *p* = 0.541; GFI > 0.974).

Stocking rate explained 68.7%, 9.1%, 44.4%, 79.1%, 81.6%, 66.1%, 90.4%, and 87.4% of the variation in CP concentration, sward height, herbage mass, ME, intake rate, bite weight, diurnal intake time, and DMI, respectively (Figure 10). The stocking rate directly reduced sward height, herbage mass, CP, and bite weight but had positive effects on ME, intake rate, and diurnal intake time. However, sward height had negative effects on CP and herbage mass; herbage mass had a positive effect on CP; ME had positive effects on diurnal intake time and intake time; CP had a positive effect on ME, intake rate, diurnal intake time, and DMI and a negative effect on bite weight. Furthermore, intake rate and bite weight directly increased DMI. 

### 3.7. Equations for Predicting Grazing Behavior 

Using CP, ADF, NDF, and ME to predict grazing behavior parameters yielded the best fit equation for Tibetan sheep, and the addition of ME for the linear relationship further improved the R^2^ of equations (Table 4).

## 4. Discussion

### 4.1. Effects of the Grazing Season and Stocking Rate on Plant Community Characteristics and the Chemical Composition of Forage

In grassland grazing ecosystems, the plant community characteristics and the chemical composition of forages were affected by stocking rate and grazing seasons [10,30]. Bear et al. [31] found that higher stocking rates had an effect on sward height. Miao et al. [32] mentioned that in the yak grazing ecosystem, forage nutritive values increased with the increase of the stocking rate, which may be due to lower mean age plant tissues, higher nutritive values of a regrowing plant, and higher nitrogen inputs from the excrement of grazing livestock at high stocking rate than that at light stocking rate. However, the result we found was inconsistent with the above views, which may be caused by the following reasons. Firstly, because forage samples were collected after 10 days of grazing in every period, the compensatory growth of forage has little effect on the plant community. Simultaneously, the experimental animals are different, and Tibetan sheep and yaks have different eating habits, Therefore, the growing plants are too short and the proportion is too low, i.e., yak generally cannot eat them, but Tibetan sheep can. Secondly, in our study, the feces of Tibetan sheep were collected, so the nutrients from grazing livestock excrement have a weak effect on pasture. Finally, we found that at a higher stocking rate, the proportion of edible forage grass is greatly reduced, while the proportion of inedible forage grass is increased, and some higher–fiber Gramineae such as *Stipa capillata Linn.*, *Elymus nutans* Griseb., and thick–stemmed *Anemone rivularis* Buch.–Ham. ex DC. var. *flore–minore* Maxim dominated the plant community.

In the present study, grazing season and stocking rate had significant effects on plant community characteristics and the chemical composition of forage, but their interaction between grazing season and stocking rate not relevant for plant community characteristics and ADF. This explained that the effects of stocking rate on plant community characteristics and ADF did not change with grazing season. Similarly, the effects of grazing season on the above indicators did not change with stocking rate. We also found that herbage mass and CP concentration of forage were higher and the ADF and NDF concentrations of forage were lower at lower stocking rate and in warm season, which illustrates the higher forage nutritive value at 8 sheep/ha in the warm season. Judy et al. [33] also found a similar result.

### 4.2. Effects of Grazing Season and Stocking Rate on Digestive and Metabolic Activities

The weight of grazing livestock always changes with the characteristics of “fat in summer and thin in winter” on the QTP. Due to the decline of forage quality (i.e., CP) and DMI in the cold season, the CPI and ADG of Tibetan sheep decreased, which led to the decrease in MEI. Liu et al. [34] also found that there was a highly linear positive correlation between the ADG of Xiangzhong black cattle and metabolic energy intake (R^2^ = 0.906). Therefore, we should supplement feed for grazing livestock, so as to obtain more nutrients to meet the nutritional requirements and normal energy metabolism in the cold season. Forage CP and herbage mass declined at higher stocking rate [35], therefore, the DMI, CPI and FDM of Tibetan sheep at 16 sheep/ha were lower than that at 8 sheep/ha.

### 4.3. Diurnal Grazing Behavior of Tibetan Sheep

In the present study, the intake rate and bite of ingestion of Tibetan sheep increased as the time of day advanced. Some research reported that, in one day, the forages had larger concentrations of total nonstructural carbohydrates and higher digestibility in the afternoon and twilight than that in the morning [36,37], which may affect the main behavior patterns [3]. In addition, ghrelin is a hormone that stimulates grazing animals’ feeding and is secreted the most at dusk and dawn [38,39]. Therefore, Tibetan sheep can obtain the maximum nutrient intake at dusk in order to ruminate at night.

De et al. [40] reported that high temperature significantly decreased the time spent in feeding, ruminating, and lying in rams. Prescott et al. [41] found that as ambient temperature increased, diurnal grazing time for beef cows increased in the cold season. Therefore, the environmental temperature can affect the behavior of livestock. However, in the present study, we found that diurnal intake rate and walking velocity while intaking increased as both daylight ambient temperature and relative humidity increased, which may be because the suitable temperature was good for Tibetan sheep to look for feed with better nutritional value, playing an important role in livestock production. Meanwhile, there was higher intake rate and lower walking speed during grazing at 16 sheep/ha than that at 8 sheep/ha, and there are statistical differences in every two regression lines, which may be due to lower herbage mass and DMI at 16 sheep/ha than that at 8 sheep/ha influencing the increased intake rate and reduced walking velocity while intaking to compensate for the lack of nutrition.

### 4.4. Effects of Stocking Rates on Grazing Behavior

High stocking rates can decrease the herbage mass available [42] and reduce the intake per bite [43] but can increase the number of steps and grazing time to compensate for the decrease of forage availability [44,45], in line with our results. In the present study, the intake per bite and walking velocity while intaking of Tibetan sheep were reduced, while intake, bite of ingestion and diurnal intake time were increased at the higher stocking rate. Here, Tibetan sheep’s intake per bite decreased, likely because sward height and herbage mass per unit area decreased at the 16 sheep/ha stocking rate. In order to obtain more nutrients to meet energy demands, under restrictive environmental conditions, Tibetan sheep have to spend more time on the intake of forage.

Ruminant activity is a significant physical process for ruminants to degrade and digest the forage, and ruminant behavior is second only to feeding behavior for the Tibetan sheep [10]. Ruminant behavior is affected by the distribution of forages and the plant composition of grasslands [46,47], as well as the quantity and quality of forages [10]. We observed that the number of chews per feed bolus, the chewing time per feed bolus, and the interval between feed boluses were reduced at the high stocking rate, which may be because of the higher ADF and NDF concentrations of forage and the lower sward height and herbage mass available at high stocking rates causing Tibetan sheep to increase diurnal intake time to obtain enough nutrients and to maintain normal metabolism, resulting in less time for ruminant activity during the day [48].

### 4.5. Effects of Grazing Season on Grazing Behavior

In the long–term coevolution process, different plant individuals from different biological clock phases in phenological phases overlap in space in order to adapt to the local living environment, which leads to the difference in plant community characteristics and chemical composition of forage. Simultaneously, the feed behavior of grazing livestock is a result of the plant community characteristics shaped by grazing animals and sward management decisions [9]. Therefore, plant community characteristics and chemical composition of forage change with the change of seasons, which affects the grazing behavior of livestock. 

In the warm season, suitable air temperature, high CP concentration, and good palatability of forage have led to an increase in intake time for Tibetan sheep. Compared with the warm season, the decreased intake rate and walking velocity while intaking in the cold season may be due to the decrease in sward height and herbage mass, the decline in forage quality, and the atrocious weather. Furthermore, the Tibetan sheep increased bite weight in the cold season, which compensated for a partial reduction in diurnal intake time. The decrease in total number of steps in the cold season could have been a way to save energy consumption in order to resist the cold environment. Blanchard et al. [49] reported that forage quality had a negative effect on the average values of rumination parameters (number of chews per feed bolus and chewing time per feed bolus), and compared to the rainy season (February), impala increased these rumination parameters in the dry season (September). We also found a similar result that compared with warm season: Tibetan sheep increased both the number of chews per feed bolus and the chewing time per feed bolus but decreased interval between boluses and ruminating rate in the cold season, which was due to the worse forage quality (higher ADF and NDF concentrations) in the cold season.

### 4.6. Effects of Grazing Season and Stocking Rate on DMI

DMI data were intended to reflect the amount of nutrients and energy that livestock obtain in their living environment and play a significant role in grazing livestock production. Our model showed that stocking rate and grazing season had direct and indirect effects on DMI. Based on the stocking rate and grazing season, the decrease in DMI was mainly due to bite weight and intake rate, which were mediated by sward height, herbage mass, CP concentration, and ME. However, the stocking rate and grazing season had significantly influenced DMI, which had higher values at a low stocking rate and in the warm season. Previous studies have shown that the seasonal change in DMI is most likely affected by the initial herbage mass available and sward height, but chemical composition of forage has no effect on DMI [50]. For instance, spring grazing initially positively affected the quality of forage and plant community structures, which in turn improved the grazing behavior characteristics of Holstein–Friesian dairy cows and increased DMI [51]. In the first model, the direct effect of grazing season on DMI was the main source of its negative effect. Seasonal changes lead to changes in the nutritional concentration of forages. At the same time, the leaves wither and fall off of the plant, which leads to a reduction in the amount of edible forages per unit area in the cold season. We found that the change of grazing season (from warm season to cold season) had a positive effect on sward height but a negative effect on DMI. This is because there were many undesirable plants, such as Gramineae, of higher ADF and NDF concentrations, harder stems, and fewer leaves in the cold season, therefore, Tibetan sheep had lower DMI in the cold season. The grazing season can also indirectly affect DMI. On the one hand, it can reduce the bite weight and thus the DMI by reducing the CP concentration of forage, and it can also reduce the intake rate of Tibetan sheep by reducing the ME to reduce the DMI. On the other hand, it can have a negative effect on herbage mass, which increases the intake rate to compensate for the lack of nutritional needs and DMI.

Chen et al. [28] found that the stocking rate had little effect on DMI, which may be due to the sward sparsely distributing on the Loess Plateau. However, this phenomenon does not exist on the QTP because of the lush herbs. In the second model, the stocking rate had a negative effect on the sward height and herbage mass, which was due to the feeding and trampling effects of Tibetan sheep. It was not difficult to determine that the stocking rate did not have a direct effect on DMI, but it can affect feeding behavior by mediating grassland condition indexes to indirectly affect DMI. In the first place, the stocking rate had a negative indirect effect on DMI by mediating bite weight, as well as a positive indirect effect on DMI by increasing bite weight due to the lack of forage CP concentration. In the second place, it had a positive indirect effect on DMI by mediating intake rate or by increasing ME to intake rate. All in all, it can be expected that Tibetan sheep have a greater opportunity to select high–quality and palatable herbage in the warm season grazing system and lower stoking rate system. Due to poor quality of forage and inadequate forage at higher stocking rate and in the cold season, Tibetan sheep had lower DMI and had difficultly into maintaining their own nutrient metabolism. Therefore, in order to prevent grassland degradation and to improve the capacity of grassland, it is feasible to sell out more Tibetan sheep to mitigate the stocking rate or to provide supplementary feeding in the cold season.

### 4.7. Prediction of Grazing Behavior Parameters

Andueza et al. [52] used the chemical composition of forage to predict DMI, and Yang et al. [25] used CP, GE, EE, NDF, and ADF parameters to predict DMI. Gross et al. [53] used the plant density and biomass to build the prediction equation for intake rate. In the current work, parameters such as CP, ADF, NDF, ME, sward height, and herbage mass appeared among the equations that can best predict grazing behavior (including DMI) for Tibetan sheep living in the northeast of the QTP. The increase in forage CP concentration, sward height, and herbage mass can increase the probability of Tibetan sheep’s selective feeding in a natural grassland. The high concentration of forage fiber (i.e., ADF and NDF) reduced the palatability and digestibility for the livestock and affected grazing behavior. However, because the addition of ME increased the R^2^ values in the above equation, ME was an indispensable predictor. Combining the above information, grazing behavior of Tibetan sheep was greatly affected by the quantity and quality of the forage.

## 5. Conclusions

This study demonstrated that the grazing behavior of Tibetan sheep was affected by grazing season and stocking rate in the alpine meadow of the QTP, China. Due to more adequate forage supply and better forage quality at low stocking rate in the warm season, Tibetan sheep had higher CPI, DMI, and total steps. Simultaneously, Tibetan sheep obtained the maximum nutrient intake at dusk by improving intake rate and the bite of ingestion. In addition, intake time and walking velocity while intaking increased as both daylight ambient temperature and relative humidity increased. Most importantly, Tibetan sheep should be added feed after grazing in the cold season to maintaining their own nutrient metabolism.

## Figures and Tables

**Table 1:** The *p*–values of the effects of grazing season, stocking rate, and their interactions on plant community characteristics and chemical composition of forage.

Variables	Sward Height	Plant Density	Herbage Mass	ADF	NDF	CP
GS	< 0.001	0.040	< 0.001	< 0.001	< 0.001	< 0.001
SR	< 0.001	< 0.001	< 0.001	< 0.001	< 0.001	< 0.001
GS×SR	0.701	0.847	0.373	0.299	< 0.001	< 0.001

Note: GS = grazing season; SR = stocking rate.

**Table 2:** The *p*–values of the effects of grazing season, stocking rate, and their interactions on the digestive and metabolic indicators of Tibetan sheep.

Variables	FDM	CPI	DMD	ME	MEI
GS	< 0.001	< 0.001	0.572	< 0.001	< 0.001
SR	< 0.001	< 0.001	0.091	< 0.001	0.401
GS×SR	0.573	< 0.001	0.679	0.044	0.096

Note: GS = grazing season; SR = stocking rate.

**Table 3:** The *p*–values of the effects of grazing season, stocking rate, and their interactions on grazing behavior of Tibetan sheep.

Variables	Diurnal Intake Time	Bite Weight	DMI	Total Number of Steps	Number of Chews per Feed Bolus	Chewing Time per Feed Bolus	Interval between Feed Boluses	Ruminating Rate
GS	< 0.001	< 0.001	< 0.001	< 0.001	< 0.001	< 0.001	0.024	< 0.001
SR	< 0.001	< 0.001	< 0.001	0.308	< 0.001	0.002	0.558	< 0.001
GS×SR	0.063	0.001	0.357	0.548	0.056	0.447	0.875	0.866

Note: GS = grazing season; SR = stocking rates.

**Table 4:** Linear prediction of grazing behavior of Tibetan sheep using plant community characteristics and chemical composition of forage and forage chemical composition parameters.

Item	Equation *	R^2^	*p*-Valve
DMI (g/day)	= 1621.896_(111.672)_−3839.527_(355.288)_ ADF + 41.531_(5.590)_ME	0.658	<0.001
Intake rate (bite/min)	= −10.668_(12.932)_ − 0.829_(0.178)_H + 69.264_(14.516)_NDF + 28.452_(44.529)_CP	0.621	<0.001
	= −4.704_(16.348)_ + 1271.701_(54941)_CP + 77.855_(16.568)_NDF − 58.2811_(26.461)_ADF − 0.021_(0.010)_HM	0.560	<0.001
	= −1.327_(15.564)_ + 260.605_(56.022)_CP + 87.88_(16.246)_NDF − 57.451_(27.105)_ADF	0.729	<0.001
	= −22.759_(14.540)_ + 346.346_(44.451)_CP + 70.883_(16.716)_NDF − 0.021_(0.010)_HM	0.528	<0.001
Bite of ingestion (bite/step)	= 0.070_(2.642)_ + 18.574_(2.972)_NDF − 0.446_(0.055)_ME − 0.007_(0.003)_HM	0.664	<0.001
	= −0.609_(2.514)_ − 0.123_(0.057)_H − 0.005_(0.003)_HM + 21.033_(6.530)_ADF + 11.177_(4.022)_NDF − 0.443_(0.053)_ME	0.712	<0.001
Walking velocity while intaking (step/min)	= 15.253_(1.233)_ − 36.999_(3.922)_ADF + 0.289_(0.062)_ME	0755	<0.001
	= −1.24_(0.814)_ + 0.368_(0.060)_ME + 71.170_(6.648)_CP	0.630	<0.001
	= 3.316_(3.485)_ − 10.850_(8.072)_ADF + 0357_(0.060)_ME + 53.447_(14.749)_CP	0.640	<0.001
Bite weight (g/bite)	= 0.027_(0.013)_ − 0.003_(0.001)_ME + 0.003_(0.001)_H − 0.074_(0.022)_NDF	0.608	<0.001
Total number of steps (step/day)	= 808.550_(477.685)_ − 98.790_(22.098)_H + 38,550.949_(3007.03)_CP + 126.855_(22.281)_ME	0.847	<0.001
	= −843.305_(341.890)_ + 46196.110_(2793.460)_CP + 118.859_(25.081)_ME	0.803	<0.001

* Units: % of dry matter (DM) for the forage chemical composition of CP, NDF, and ADF; MJ/kg of DM for forage ME; cm for sward height (H); g of DM for herbage mass (HM). Values in subscripted parentheses represent SE.

## References

[1] Fonseca L., Mezzalira J.C., Bremm C., Filho R.S.A., Gonda H.L., Carvalho P.C.F. (2012). Management targets for maximising the short–term herbage intake rate of cattle grazing in Sorghum bicolor. Livest. Sci..

[2] Lkhagva A., Boldgiv B., Goulden C.E., Yadamsuren O., Lauenroth W.K. (2013). Effects of grazing on plant community structure and aboveground net primary production of semiarid boreal steppe of northern Mongolia. Grassl. Sci..

[3] Mario C.H., Nicole W.M., Johannes I. (2017). Behavioral patterns of (co–)grazing cattle and sheep on swards differing in plant diversity. Appl. Anim. Behav. Sci..

[4] Sharpe P., Kenny L.B., Sharpe P. (2019). Grazing Behavior, Feed Intake, and Feed Choices. Horse Pasture Management.

[5] Friday O.Z., Joseph O.A., Peter I.R., Muhammed U.K., Ndazo S.M., Folashade O., Mohammed J.I., Daniel O.A. (2018). Daily rhythmicity of behavioral responses in donkeys of different age groups during the cold–dry (harmattan) and hot–dry season in a tropical savannah. J. Vet. Behav..

[6] Carlos A.S.C., Juan T.G., Juan Felipe J.T.A., Pedro G.G.P. (2015). Feeding behavior of sheep and goats in a deciduous tropical forest during the dry season:The same menu consumed differently. Small Ruminant Res..

[7] Enri S.R., Gorlier A., Nota G., Pittarello M., Lombardi G., Lonat M. (2019). Distance from Night Penning Areas as an Effective Proxy to Estimate Site Use Intensity by Grazing Sheep in the Alps. Agronomy.

[8] Venter Z.S., Hawkins H.J., Cramer M.D. (2019). Cattle don’t care: Animal behaviour is similar regardless of grazing management in grasslands. Agr. Ecosyst. Environ..

[9] Hirata M., Kunieda E., Tobisa M. (2010). Short–term ingestive behaviour of cattle grazing tropical stoloniferous grasses with contrasting growth forms. J. Agr. Sci.

[10] Du W.C., Yan T., Chang S.H., Wang Z.F., Hou F.J. (2017). Seasonal hogget grazing as a potential alternative grazing system for the Qinghai–Tibetan Plateau: Weight gain and animal behaviour under continuous or rotational grazing at high or low stocking rates. Rangeland J..

[11] Lin L.J., Dickhoefer U., Müller K., Wurina, Susenbeth A. (2011). Grazing behavior of sheep at different stocking rates in the Inner Mongolian steppe, China. Appl. Anim. Behav. Sci..

[12] Teixeira D.L., Carlos P.M.F.L., Hötzel M.J., Enríquez-Hidalgo D. (2017). Effects of instantaneous stocking rate, paddock shape and fence with electric shock on dairy cows’ behaviour. Livest. Sci..

[13] Carvalho P.C.F. Can grazing behaviour support innovations in grassland management. Proceedings of the 22nd International Grassland Congress.

[14] Fabian Y., Sandau N., Bruggisser O.T., Kehrli P., Aebi A., Rohr R.P., Bersier L.F. (2012). Diversity protects plant communities against generalist molluscan herbivores. Ecol. Evol..

[15] Wrage N., Strodthoff J., Cuchillo H.M., Isselstein J., Kayser M. (2011). Phytodiversity of temperate permanent grasslands: Ecosystem services for agriculture and livestock management for diversity conservation. Biodivers. Conserv..

[16] Ren J.Z., Hu Z.Z., Zhao J., Zhang D.J., Hou F.J., Li H.L., Mu X.D. (2008). A grassland classification system and its application in China. Rangeland J..

[17] Sun Y., Angerer J.P., Hou F.J. (2015). Effects of grazing systems on herbage mass and liveweight gain of Tibetan sheep in eastern Qinghai–Tibetan Plateau, China. Rangeland J..

[18] Ma T., Chen D.D., Tu Y., Zhang N.F., Si B.W., Deng K.D., Diao Q.Y. (2015). Effect of dietary supplementation with resveratrol on nutrient digestibility, methanogenesis and ruminal microbial flora in sheep. J. Anim. Physiol An. N..

[19] Goering K.H., Van Soest P.J. (1970). Forage Fiber Analysis (Apparatus, Reagents, Procedures, and Some Application).

[20] Cunniff P.A. (1995). Official Methods of Analysis of AOAC International. Aoac. Official Method.

[21] Davis M.P., Freetly H.C., Kuehn L.A., Wells J.E. (2014). Influence of dry matter intake, dry matter digestibility, and feeding behavior on body weight gain of beef steers. J. Anim. Sci..

[22] Hou F.J., Li G., Yang F.G. (2003). Grazing behavior of Gansu wapiti (Cervus elaphus kansuensis) in summer & winter on the alpine grasslands of Qilianshan Mountain. Acta. Ecologica. Sinica..

[23] Smit H.J., Taweel H.Z., Tas B.M., Tamminga S., Elgersma A. (2005). Comparison of techniques for estimating herbage intake of grazing dairy cows. J. Dairy Sci..

[24] Agricultural and Food Research Council (AFRC) (1993). Energy and Protein Requirements of Ruminants.

[25] Yang C.T., Gao P., Hou F.J., Yan T., Chang S.H., Chen X.J., Wang Z.F. (2018). Relationship between chemical composition of native forage and nutrient digestibility by Tibetan sheep on the Qinghai–Tibetan Plateau. J. Anim. Sci..

[26] Koong L.J., Ferrell C.L., Nienaber J.A. (1985). Assessment of interrelationships among levels of intake and production, organ size and fasting heat production in growing animals. J. Nutr..

[27] Degen A.A., Young B.A. (2002). Effect of air temperature and energy intake on body mass, body composition and energy requirements in sheep. J. Agric. Sci..

[28] Chen X.J., Hou F.J., Matthew C., He X.Z. (2010). Stocking rate effects on metabolizable energy intake and grazing behaviour of Tan sheep in steppe grassland on the Loess Plateau of Northwest China. J. Agr. Sci..

[29] Grace J.B. (2006). Structural Equation Modeling and Natural Systems.

[30] Aharoni Y., Brosh A., Orlov A., Shargal E., Gutman M. (2004). Measurements of energy balance of grazing beef cows on Mediterranean pasture, the effects of stocking rate and season: 1. Digesta kinetics, faecal output and digestible dry matter intake. Livest. Prod. Sci..

[31] Bear D.A., Russell J.R., Tufekcioglu M., Isenhart T.M., Morrical D.G., Kovar J.L. (2012). Stocking rate and riparian vegetation effects on physical characteristics of riparian zones of Midwestern Pastures. Rangeland Ecol. Manag..

[32] Miao F.H., Guo Z.G., Xue R., Wang X.Z., Shen Y.Y. (2015). Effects of Grazing and Precipitation on Herbage Biomass, Herbage Nutritive Value, and Yak Performance in an Alpine Meadow on the Qinghai–Tibetan Plateau. PloS ONE.

[33] Judy J.V., Jenkins K.H., Klopfenstein T.J., Stalker L.A., Volesky J.D. (2015). Effects of stocking rate on forage nutrient composition of Nebraska Sandhills upland range when grazed in early summer1. J. Anim Sci..

[34] Liu D.M., Fu D.B., Qu M.R., Zhu Y.K., Li F.Q., Deng X.T. (2013). Energy metabolism and requirement of 12 to 13–month–old Xiangzhong black cattle. Chinese J. Ani. Nutri..

[35] Jung H.G., Sahlu T. (1889). Influence of grazing pressure on forage quality and intake by sheep grazing smooth bromegrass. J. Anim. Sci..

[36] Edouard N., Duncan P., Dumont B., Baumont R., Fleurance G. (2010). Foraging in a heterogeneous environment–An experimental study of the trade–off between intake rate and diet quality. Appl. Anim. Behav. Sci..

[37] Villalba J.J., Provenza F.D., Manteca X. (2010). Links between ruminants´ food preference and their welfare. Animal.

[38] Roche J.R., Sheahan A.J., Chagas L.M., Boston R.C. (2008). Short communication: change in plasma ghrelin in dairy cows following an intravenous glucose challenge. J. Dairy Sci..

[39] Gregorini P., Soder K.J., Kensinger R.S. (2009). Effects of rumen fill on short–term ingestive behavior and circulating concentrations of ghrelin, insulin, and glucose of dairy cows foraging vegetative micro–swards. J. Dairy Sci..

[40] De K., Kumar D., Saxena V.K., Thirumurugan P., Naqvi S.M.K. (2017). Effect of high ambient temperature on behavior of sheep under semi–arid tropical environment. Int. J. Biometeoro..

[41] Prescott M.L., Havstad K.M., Olson-Rutz K.M., Ayers E.L., Petersen M.K. (1994). Grazing behavior of free–ranging beef cows to initial and prolonged exposure to fluctuating thermal environments. Appl. Anim. Behav. Sci..

[42] Wang R.Z. (2004). Responses of Leymus chinensis (Poaceae) to long–term grazing disturbance in the Songnen grasslands of north–eastern China. Grass Forage Sci..

[43] Forbes T.D.A. (1988). Researching the plant–animal interface: the investigation of ingestive behavior in grazing animals. J. Anim. Sci..

[44] Animut G., Goetsch A.L., Aiken G.E., Puchala R., Detweiler G., Krehbie C.R., Merkel R.C., Sahlu T., Dawson L.J., Johnson Z.B. (2005). Grazing behavior and energy expenditure by sheep and goats co–grazing grass/forb pastures at three stocking rates. Small Ruminant Res..

[45] Augustine D.J., Springer T.L. (2013). Competition and facilitation between a native and a domestic herbivore: trade–offs between forage quantity and quality. Ecol. Appl..

[46] Liu J., Feng C., Wang D., Wang L., Wilsey B.J., Zhong Z.W. (2015). Impacts of grazing by different large herbivores in grassland depend on plant species diversity. J. Appl. Ecol..

[47] Ferreira L.M.M., Hervás G., Belenguer A., Celaya R., Rodrigues M.A.M., García U., Frutos P. (2017). Comparison of feed intake, digestion and rumen function among domestic ruminant species grazing in upland vegetation communities. J. Anim. Physiol An. N..

[48] Hai L., Ao T.G., Wang C.J., A L.M.S., San R.G.W. (2012). Effects of different grazing intensities on herding behavior of Simmental cattle. Grassland and Prataculture. Grassland and Prataculture.

[49] Blanchard P., Fritz H. (2008). Seasonal variation in rumination parameters of free–ranging impalas Aepyceros melampus. Wildlife Biol..

[50] Dowler L.E., Siciliano P.D., Pratt-Phillips S.E., Poore M. (2012). Determination of pasture dry matter intake rates in different seasons and their application in grazing management. J. Equine. Vet. Sci..

[51] Kennedy E., O’Donovan E., Murphy J.P., Delaby L., O’Mara F.P. (2007). Effect of spring grazing date and stocking rate on sward characteristics and dairy cow production during midlactation. J. Dairy Sci..

[52] Andueza D., Picard F., Pradel P., Egal D., Hassoun P., Peccatte J.R., Baumont R. (2011). Reproducibility and repeatability of forage in vivo digestibility and voluntary intake of permanent grassland forages in sheep. Livest. Sci..

[53] Gross J.E., Hobbs N.T., Wunder H.B.A. (1993). Independent variables for predicting intake rate of Mammalian Herbivores: biomass density, plant density, or bite size. Oikos.

